# Comparative Analysis of DNA Methylation Reveals Specific Regulations on Ethylene Pathway in Tomato Fruit

**DOI:** 10.3390/genes9050266

**Published:** 2018-05-21

**Authors:** Jinhua Zuo, Yunxiang Wang, Benzhong Zhu, Yunbo Luo, Qing Wang, Lipu Gao

**Affiliations:** 1 Key Laboratory of the Vegetable Postharvest Treatment of Ministry of Agriculture, Beijing Vegetable Research Center, Beijing Academy of Agriculture and Forestry Sciences, Beijing 100097, China; 2 Beijing Key Laboratory of Fruits and Vegetable Storage and Processing, Beijing Vegetable Research Center, Beijing Academy of Agriculture and Forestry Sciences, Beijing 100097, China; 3 Key Laboratory of Biology and Genetic Improvement of Horticultural Crops (North China) of Ministry of Agriculture, Beijing Vegetable Research Center, Beijing Academy of Agriculture and Forestry Sciences, Beijing 100097, China; 4 Key Laboratory of Urban Agriculture (North) of Ministry of Agriculture, Beijing Vegetable Research Center, Beijing Academy of Agriculture and Forestry Sciences, Beijing 100097, China; 5 Boyce Thompson Institute for Plant Research, Cornell University Campus, Ithaca, NY 14853, USA; 6 Beijing Academy of Forestry and Pomology Sciences, Beijing Academy of Agriculture and Forestry Sciences, Beijing 100093, China; yunxiangjkkl@126.com; 7 Laboratory of Postharvest Molecular Biology of Fruits and Vegetables, Department of Food Biotechnology, College of Food Science and Nutritional Engineering, China Agricultural University, Beijing 100083, China; zbz@cau.edu.cn (B.Z.); lyb@cau.edu.cn (Y.L.)

**Keywords:** DNA methylation, specific regulations, ethylene pathway, tomato fruit

## Abstract

DNA methylation is an essential feature of epigenetic regulation and plays a role in various physiological and biochemical processes at CG, CHG, and CHH sites in plants. LeERF1 is an ethylene response factor (ERF) found in tomatoes which plays an important role in ethylene signal transduction. To explore the characteristics of DNA methylation in the ethylene pathway, sense-/antisense-LeERF1 transgenic tomato fruit were chosen for deep sequencing and bioinformatics parsing. The methylation type with the greatest distribution was CG, (71.60–72.80%) and CHH was found least frequently (10.70–12.50%). The level of DNA methylation was different among different tomato genomic regions. The differentially methylated regions (DMRs) and the differentially expressed genes (DEGs) were conjointly analyzed and 3030 different expressed genes were found, of which several are involved in ethylene synthesis and signaling transduction (such as *ACS*, *ACO*, *MADS-Box*, *ERFs*, and *F-box*). Furthermore, the relationships between DNA methylation and microRNAs (miRNAs) were also deciphered, providing basic information for the further study of DNA methylation and small RNAs involved in the ethylene pathway.

## 1. Introduction

DNA methylation is a type of epigenetic modification process that is necessary for the control of genome activity in animals and plants [1]. DNA methylation exists in three sequence contexts in plants (mCG, mCHG, and mCHH), each involving different mechanisms for the establishment and maintenance of DNA methylation [2,3,4]. In regard to the symmetrical methylation types, histone methyltransferases are required for DNA methylation; CG methylation is catalyzed by methyltransferase1 (MET1) and CHG methylation is set up mainly by the plant-specific enzyme, chromomethylase 3 (CMT3) [5,6,7]. For methylation at nonsymmetrical (CHH) sites, two important enzymes assigned as domains rearranged methyltransferases (DRM1 and DRM2) are in charge [8,9]. In this process, methylation is guided by small (typically 24 nucleotide) RNAs known as small interfering RNAs (siRNAs) [9,10]. DNA methylation is largely considered to function in the transcriptional silencing of transposable elements (TEs) to maintain genome stability and integrity as well as possibly regulating specific genes, such as those subjected to genomic imprinting [4,11,12,13,14].

The tomato is a model system used in the study of fruit ripening and the ethylene pathway during senescence because of its genetic and molecular tractability [15,16]. The tomato fruit ripening process can be divided into several distinct developing stages and in ripening it becomes a complicated process with variation in flavor, color, texture, and nutrition [17,18]. Ethylene plays important roles in developmental and physiological regulation in various higher plants whose effects are more distinctive during the ripening process of climacteric fruit [19]. Ethylene is an initiator of tomato fruit ripening and reducing its biosynthesis or interfering with its perception inhibits the ripening process [20]. Although the regulation mechanisms of fruit ripening by ethylene have not been fully deciphered, they are known to be sophisticated [21,22,23]. It has been reported that the ethylene signal transduction regulated genes are mediated by ethylene response factors (ERFs) [24]. Ethylene response factors are one of the largest families of plant transcription factors and a total of 146 members in the tomato AP2/ERF superfamily have been identified in a whole genome, of which 77 members belong to the ERF family [24,25]. Expression profiling analyses and functional studies have shown that numerous tomato ERF genes influence growth regulation and developmental processes, such as seed germination and development, flower pedicel abscission, fruit ripening, and senescence [24,26,27,28].

ERF1 is an important transcription factor that plays important roles in the regulation of downstream ethylene responsive genes through binding to the ‘GCC’ box promoter element, a conserved sequence of ethylene response genes [16,18,29]. Furthermore, recent studies have indicated that ERF1 may participate in the coordination of phenolics accumulation in tomato fruit and fruit ripening [30]. In the current study, to explore the specific characteristics of DNA methylation, ethylene biosynthesis and signal transduction in tomato fruit, sense-/antisense-LeERF1 transgenic tomatoes were sequenced and a further bioinformatics analysis was completed. Distribution varied between methylation types and methylation levels varied between tomato genomic regions. The differentially methylated regions (DMRs) and differentially expressed genes (DEGs) were analyzed conjointly, and several genes were found to be involved in the ethylene pathway, such as ACS, ACO, MADS-Box, and ERFs. In addition, the relationships between DNA methylation and micro RNAs (miRNAs) were deciphered. This research expands the understanding of the regulatory pathways of DNA methylation in the network of ethylene pathways.

## 2. Materials and Methods

### 2.1. Sample Collection and Library Generation for Bisulfite-Seq

Control (*Solanum lycopersicum* cv. Zhongshu4) and sense-/antisense-LeERF1 transgenic tomato plants were grown in a greenhouse. Tomato fruit from each of the three groups at the breaker stage were picked and transported to the lab for experimental preparation, and the pericarp tissues were quickly frozen in liquid nitrogen and stored at −80 °C for the next step. Bisulfite-seq (BS-seq) libraries were made from genomic DNA isolated from these tissues. DNA was first fragmented by sonication to 100 to 300 base pair (bp) in size, followed by end-blunting, dA addition at the 3′ end, and ligation of adapters. Next, adaptor-ligated molecules of 200 to 300 bp were isolated by agarose gel electrophoresis and subjected to a treatment of sodium bisulfite conversion using the ZYMO EZ DNA Methylation-Gold kit (ZYMO Research Corporation, Irvine, California, USA). Finally, the Polymerase chain reaction (PCR) enriched libraries were purified and subjected to high-throughput sequencing in BioMarker (Biomarker Technologies Co., Beijing, China).

### 2.2. Quality Control

Raw data (raw reads) in the FASTQ format were first processed through in-house Perl scripts. In this step, clean data (clean reads) were obtained by removing low quality reads and reads containing adapters or ploy-N. At the same time, the Q20, Q30, GC-content, and sequence duplication level of the clean data were calculated. All the downstream analyses were based on the clean, high quality data.

### 2.3. Methylation Calling

Sequences were aligned to the reference genome using the Bismark aligner (v0.7.0) [31] under the parameters-N 1, -L 20 and -bowtie2. Methylated cytosines were extracted from the aligned reads using the Bismark methylation extractor under standard parameters (Supplied by BioMarker).

### 2.4. Methylation Level

A binomial test was used to determine if the observed methylation frequency was above the background expected from inefficiencies in the bisulfite conversion reaction and sequencing errors. When using this test (false discover rate (FDR) < 0.05), the amount of methylation at a given site is typically expressed as the ratio of reads with methylation out of the total number of reads covering the position (≥2x). We refer to this site-specific metric as the methylation level of the site [32].

### 2.5. Detection of Differentially Methylated Regions

We used ComMet (v1.1) (a part of the Bisulfighter package) to detect differentially methylated regions. DMR detection was done using a two-step procedure. First, differentially methylated cytosines (DMCs) were detected through a comparison of alignment results between the samples. Then, DMCs at neighboring positions were grouped as contiguous DMRs using certain distance criteria. ComMet detects DMRs based on log-likelihood ratio scores (-threshold zero for CG, -noncpg -threshold 30 for CHG and CHH). The score for detecting a certain region (no change: NoCh) as a DMR directed to dir (=UP or DOWN) was defined using MOABS (Model-based Analysis of Bisulfite Sequencing data), based on a β-binomial hierarchical model. The regions where the Fisher’s exact test *p*-value was less than 0.05 were recognized as DMRs.

### 2.6. Integrated Functional Analysis of Differentially Metilated Genes

Genes were compared against various protein databases by Basic Local Alignment Search Tool (BLAST)X including the National Center for Biotechnology Information (NCBI) non-redundant protein (Nr) database (ftp://ftp.ncbi.nih.gov/blast/db/)and the Swiss-Prot database (http://www.uniprot.org/), with a cut-off E-value of 10^-5^. Furthermore, we used KEEG Orthology Based Annotation System (KOBAS) to examine the Kyoto Encyclopedia of Genes and Genomes (KEGG) pathways for genes. Genes were retrieved based on the best BLAST hit (highest score), along with their protein functional annotation [33]. To annotate the gene in gene ontology (GO) terms, the Nr BLAST results were imported into the Blast2GO program [34]. GO annotations for the genes were obtained by Blast2GO. This analysis mapped all of the annotated genes to GO terms in the database and counted the number of genes associated with each term. The Perl script was then used to plot the GO functional classification for the UniGenes with a GO term hit to view the distribution of gene functions [35]. The obtained annotation was enriched and refined using TopGo (R package) with the “elim” method and the Kolmogorov–Smirnov test [36]. The gene sequences were also aligned to the Clusters of Orthologous Group (COG) database [37] to predict and classify functions. KEGG pathways were enriched using the right-sided Fisher’s exact test.

### 2.7. DNA Methylation and MicroRNAs’ Targets Analysis

Targeted genes of selected miRNAs are listed, then the methylation levels of corresponding genes were calculated according to the results of methylation analysis [32].

## 3. Results

### 3.1. DNA Methylation Landscapes in Tomato Fruit

**Figure 1:**
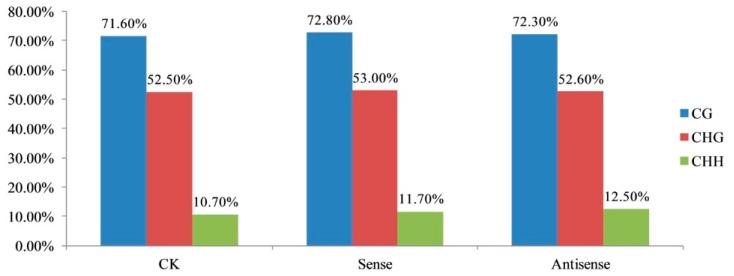
The methylation levels of different samples. The methylation levels of CK (control), sense, and antisense tomato fruit were analyzed, the CG type showed the greatest level of methylation, from 71.6 to 72.8%, followed by CHG type with 52.5 to 53%, and then CHH type with 10.7 to 12.5%.

To investigate the specific regulations of DNA methylation on the ethylene pathway, CK and sense-/antisense-LeERF1 transgenic tomato fruit were sequenced and analyzed. After data filtering and mapping to the tomato genome, 99.99, 108.32, and 116.34 million unique mapped reads remained in the CK (control), sense, and antisense samples for further analysis, respectively (Tables S1 and S2). We analyzed the methylation reads of mCG, mCHG, and mCHH, which reflected the methylation levels of mCs in the three samples. As shown in Figure 1, methylcytosine was most often found at the CG sites (71.60, 72.80, and 72.30%) in the CK, sense and antisense samples, respectively, with less frequent occurrences in the CHG and CHH sequences (52.50, 53.00, 52.60%; and 10.70, 11.70, 12.50%, respectively). This indicates that the CG type had the greatest level of methylation (Figure 1, Table S3).

**Figure 2:**
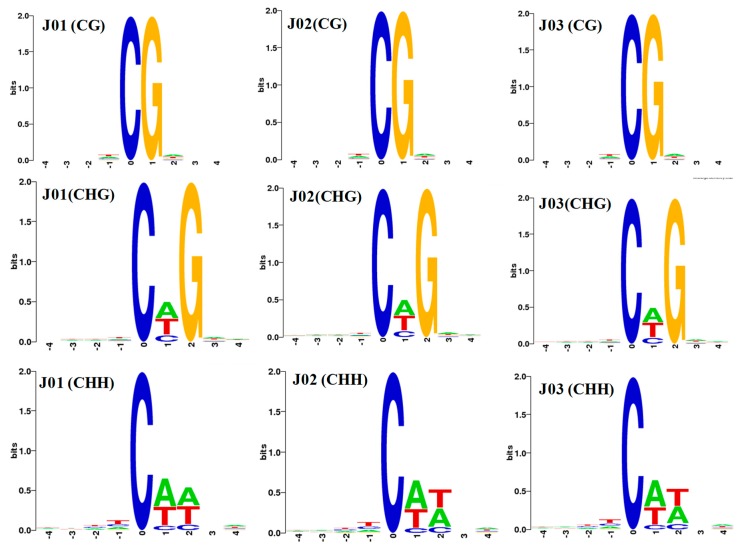
The methylation preferences in nine base pairs (bp) spanning methylcytosine sites. CG was the most common sequence motif at the CG mC sites (first row), CAG was the most common sequence motif at the CHG mC sites (second row), and CAT and CAA were the most common sequence motifs at the CHH mC sites (third row). The abscissa is the base number of the methylation site, and the total height of each position is the sequence conservation of the base, which represents the relative frequency of the base at that position.

In addition, we analyzed the relationship between the sequence context and methylation preference. We analyzed the percentage of methylation for all possible 9-mer sequences. CG was the most common sequence motif at the CG mC sites. CAG was the most common sequence motif at the CHG mC sites, and CAT and CAA were the most common sequence motifs at the CHH mC sites. Different frequencies of CHH were found for the two groups (Figure 2). 

### 3.2. DNA Methylation Patterns in Different Tomato Genomic Regions

**Figure 3:**
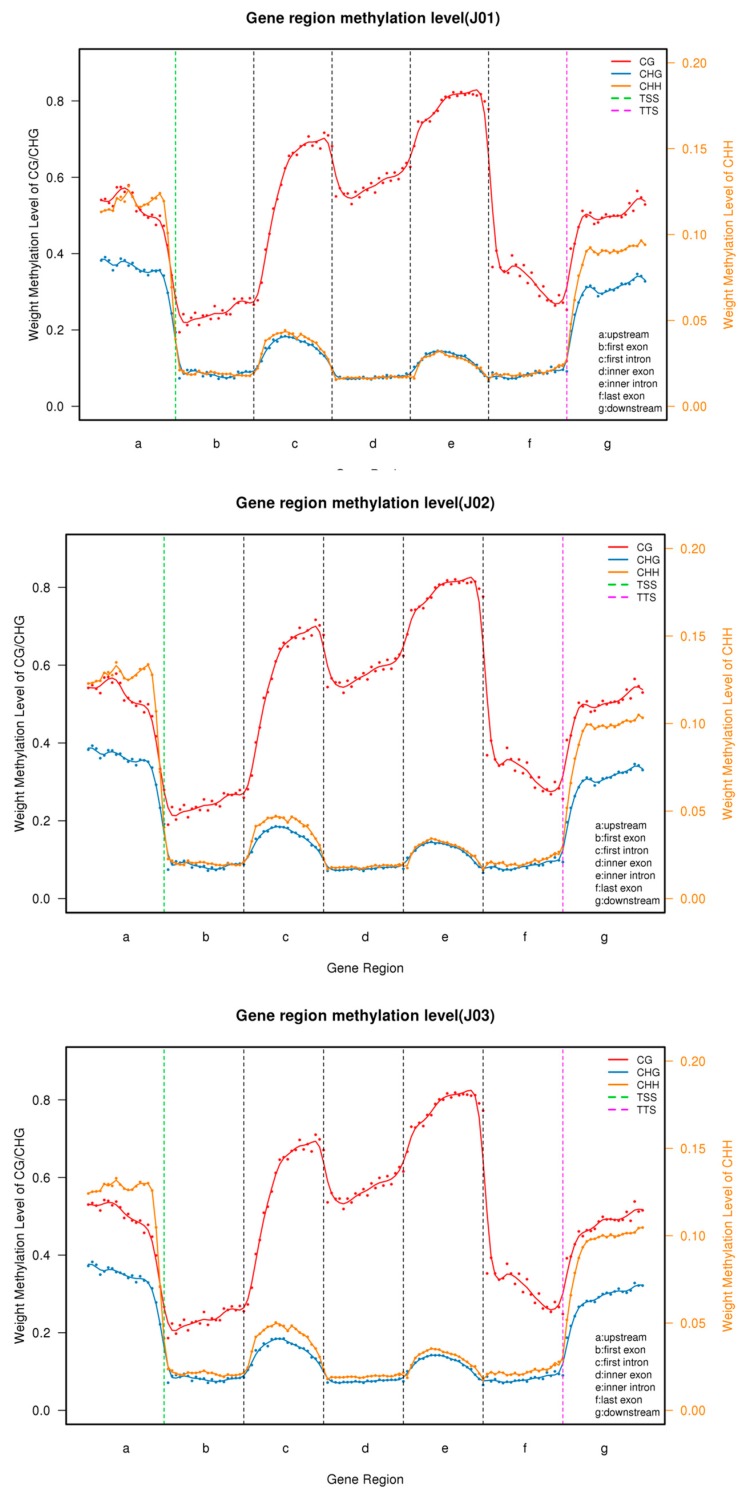
The DNA methylation levels of different gene regions of different samples. The methylation levels of the different gene regions from the different samples showed the same trends. The CG type was found more frequently than the CHG and CHH types, which showed almost the same level of occurrence. J01 represents the CK, J02 represents the sense, and J03 represents the antisense samples. TSS: Transcriptional Start Site; TTS: Transcriptional Termination Site.

**Figure 4:**
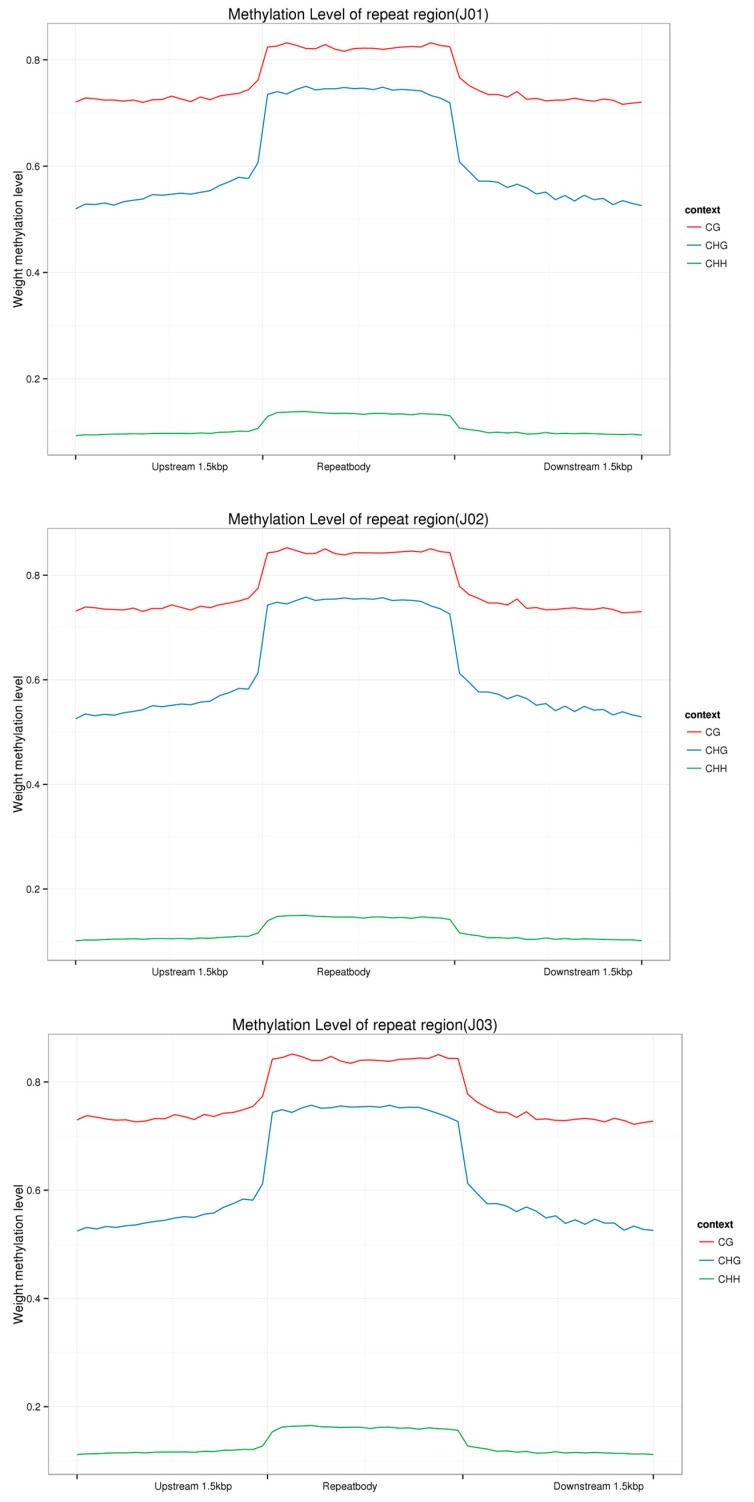
The methylation levels of the repeat regions of different samples. The CG, CHG, and CHH types were first raised and decreased in the downstream region and the methylation levels of the CG and CHG type were considerably higher than the CHH type in the CK (J01), sense (J02), and antisense samples (J03).

To explore the DNA methylation patterns in different tomato genomic regions, we analyzed the methylation profiles within genes. In the upstream region, the CHH type was found slightly more frequently than the CG type except for in the control tomatoes, but was found more frequently than the CHG type in all three samples. In the first intron, inner intron, last intron, first exon, inner exon, and last exon region (from the transcription start site to the transcription terminal site), the CG type was found more frequently than the CHG and CHH types, which showed almost the same level of occurrence. However, in the downstream region, CG was found most frequently, followed by CHH and then CHG (Figure 3). In addition, methylation levels were analyzed in the repeat region where CG, CHG, and CHH were first raised and then decreased in the downstream region. The methylation levels of the CG and CHG types were considerably higher than that of the CHH type (Figure 4).

**Figure 5:**
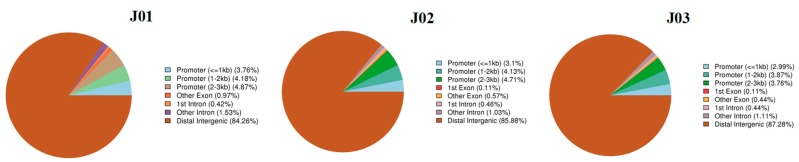
The annotation of the hypermethylated CpG islands (CGI) regions in different samples. More than 84% of the hypermethylated CGI regions were distributed in the distal intergenic regions and about 10% were distributed in the promoter region.

We calculated the quantity of hypermethylated CGI regions (CpG islands) and annotated these with gene functional elements. More than 84% of the hypermethylated CGI regions were distributed in the distal intergenic regions and about 10% were distributed in the promoter region. The CGI region of the promoter (2–3 kb) in the antisense samples was smaller than that in the control and the sense samples. In addition, the CGI region in the other exons of the control samples was larger than that in the sense and antisense samples, and there was no remarkable difference among the three groups in the other gene functional elements (Figure 5).

### 3.3. Differentially Methylated Regions Analysis and Function Parsing

**Figure 6:**
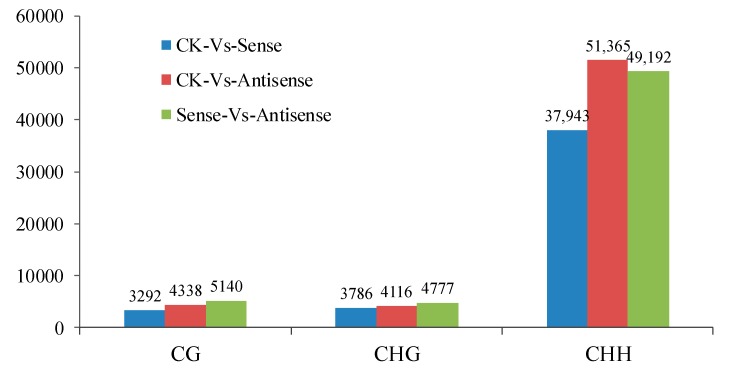
The differentially methylated regions (DMRs) of the different methylation types in different samples. Numerous DMRs were detected in the three comparative groups—3292, 4338, and 5140 DMRs were found in the CG methylation type; and 3786; 4116; and 4777 DMRs and 37,943; 51,365; and 49,192 DMRs were found in the CHG and CHH methylation types, respectively.

Numerous DMRs were detected in the three comparative groups and were annotated into gene functional elements according to different methylation types (Figure 6). The length distributions of the DMRs of different chromosomes were also calculated (Figures S1–S3). We analyzed the methylated genes in the CK_vs_Sense, CK_vs_Antisense, and Sense_vs_Antisense samples, and the topGO results of the three methylation types (CG, CHG, and CHH) varied in regard to biological processes, cellular components, and molecular functions, which indicated that DNA methylation could play important roles in the ethylene pathway (Table S4).

**Figure 7:**
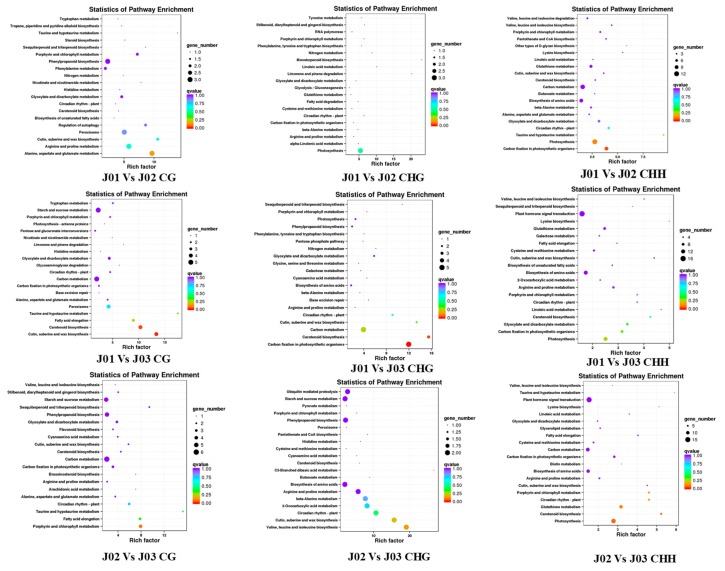
The KEGG pathway analysis of the DMRs in the different methylation types and different comparative groups. For the three methylation types (CG, CHG, and CHH) in the methylated genes of the CK_vs_Sense and CK_vs_Antisense groups, the pathway mainly focused on oxidative phosphorylation, starch and sucrose metabolism, and plant hormone signal transduction. In contrast, the methylated genes of the Sense_vs_Antisense groups of the CHG methylation type were different and mainly included the pentose phosphate pathway, limonene and pinene degradation, and protein export.

Compared with the GO results, the KEGG results were different and had more variety. For the three methylation types (CG, CHG, and CHH) in the methylated genes of the CK_vs_Sense and CK_vs_Antisense groups, the pathway mainly focused on oxidative phosphorylation, starch, and sucrose metabolism and plant hormone signal transduction. In contrast, the functions of the methylated genes of the Sense_vs_Antisense groups of the CHG methylation type were different and mainly included the pentose phosphate pathway, limonene and pinene degradation, and protein export (Table S4). In addition, the functions of the methylated genes of the Sense_vs_Antisense groups of the CHH methylation type were also different and mainly included phagosome, arachidonic acid metabolism, and pyruvate metabolism (Figure 7, Table S4).

### 3.4. Differentially Methylated Regions and Differentially Expressed Genes Conjoint Analysis Involved in the Ethylene Pathway 

The conjoint analysis of DMRs and DEGs in DNA methylation identified 3030 differently expressed genes. For the CG, CHG, and CHH methylation types, the top GOs of the methylated genes of the CK_vs_Sense, CK_vs_Antisense, and Sense_vs_Antisense comparative groups revealed different regulations for the various molecular functions and biological processes (Table S5). 

**Figure 8:**
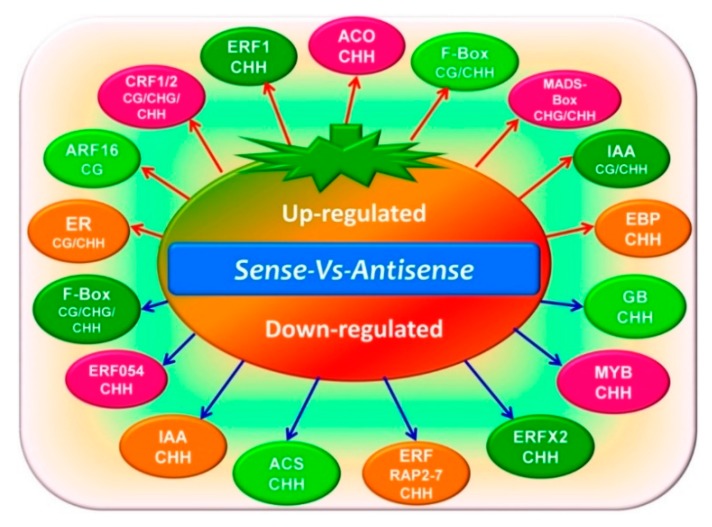
The DMR and differentially expressed genes (DEG) conjoint analysis of related genes involved in the ethylene pathway. Several genes of different methylation types involved in ethylene synthesis and signal transduction were identified.

Compared with the GO results, the KEGG results showed more differences between the three methylation types (CG, CHG, and CHH) and the separate comparable groups (CK_vs_Sense, CK_vs_Antisense, and Sense_vs_Antisense). For the CG methylation type, the methylated genes were found to be involved in several different pathways such as carbon metabolism and starch and sucrose metabolism. Intriguingly, in the DMR and DEG conjoint analysis, particularly in the Sense_vs_Antisense group, several genes were found to be involved in the ethylene synthesis and signaling pathway (such as *ACS*, *ACO*, *MADS-Box*, *ER*, *ERFs*, and *F-box*). In addition, the genes involved in other hormone and transcription factors (*IAA*, *GB*, *ARF*, and *MYB*) were also found, which indicates the potential significant roles of DNA methylation in the ethylene pathway (Figure 8, Table S6). 

### 3.5. DNA Methylation and microRNAs

**Figure 9:**
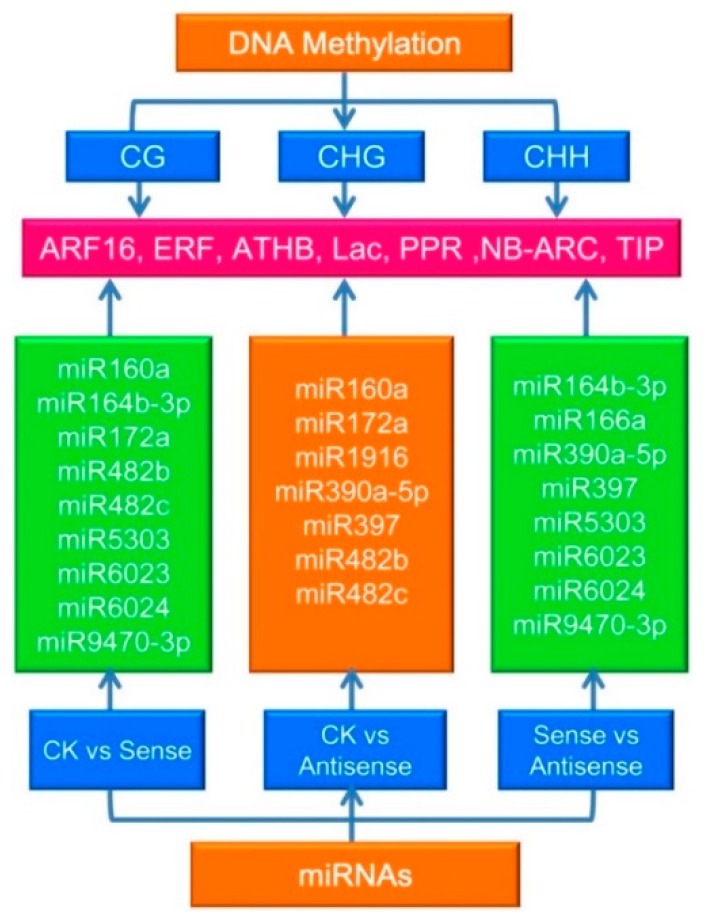
The DNA methylation and microRNA (miRNA) conjoint analysis of different groups. One hundred and ninety-one target genes with different levels of methylation were found. The DEGs of the miRNAs were analyzed and 9, 7, and 8 differently expressed miRNAs were found for each group, respectively.

MicroRNAs play vital roles in plant development and fruit ripening and senescence. Previous studies have paid more attention to the gene-repression mediated by miRNAs. In this work, the relationship between the DNA methylation pattern and levels of miRNA target genes was deciphered and191 target genes with different levels of methylation were found. The DEGs of the miRNAs between CK_vs_Sense, CK_vs_Antisense, and Sense_vs_Antisense groups were analyzed and the targets of the DEG miRNAs were analyzed. Among the CK_vs_Sense, CK_vs_Antisense, and Sense_vs_Antisense samples, 9, 7, and 8 different expressed miRNAs were found, respectively. The target genes were involved in *ARF16*, *ERF*, *ATHB*, *Lac*, *PPR*, *NB-ARC*, and *TIP* (Figure 9, Table S7). 

## 4. Discussion

Most of the tomato *ERF* genes identified to date are ethylene inducible and have ripening-related expression [38,39]. ERF1 is an important ethylene transcription factor that plays vital roles in fruit ripening in tomatoes [16,30,40]. Recently, numerous studies have focused on identifying the genome-wide methylation patterns and the functions of all kinds of abiotic and biotic stresses in plants, but few have reported on fruit and vegetables [9,29]. In the current study, we used WGBS to investigate the DNA methylation patterns of the genome in sense-/antisense-LeERF1 transgenic tomato fruit to explore the relationship between DNA methylation and the ethylene pathway. Several key enzymes and transcriptional factors—such as ACS, ACO, and ERF—showed distinct expression levels for different methylation types between the three groups. This revealed links between DNA methylation and ethylene in tomato fruit.

The traditional classification of DNA methylation in terms of CG, CHG, and CHH sequences in plant genomes indicates the functions of different DNA methyltransferases related to the establishment and maintenance of epigenetic marks [41,42]. Here, we analyzed the single-base tomato fruit methylome and the methylcytosine most often found at CG sites (71.60–72.30%). There were less frequent occurrences in the CHG and CHH sequences (52.50–53.00% and 10.70–12.50%, respectively), which indicates that the CG type is the principal sequence in tomato fruit. This finding is consistent with previous studies. The reason why the methylation pattern was a bit different may be due to the different fruit varieties and ripening stages that have been used [21]. In previous studies, CG methylation revealed the highest levels, ranging from ~30.5% in *Arabidopsis* to ~92.5% in *Beta vulgaris* [9]. CHG and CHH methylation varied from ~9.3% in *Eutrema salsugineum* to ~81.2% in *Beta vulgaris*, and ~1.1% in *Vitis vinifera* to ~18.8% in *Beta vulgaris* [3,9]. In addition, CAG was the most common sequence motif at the CHG mC sites, as also found in previous studies [29,43], and the sequence motif in CHH differed between the two groups. 

The methylome has been studied in the ethylene mutants *rin* and *nor* in tomato fruit and more than 60% of its genome contains heavily methylated transposable elements, which are concentrated in the pericentromeric heterochromatin regions [21,44]. The gene-rich euchromatic regions are located in the chromosome arms and are distinguished by a reduced methylcytosine density and methylation rates [21]. In the current study, approximately 84% of hypermethylated CGI was distributed in the distal intergenic regions and about 10% was distributed in the promoter regions. In addition, in the promoter region, the CHH type was slightly more frequent than the CG type, except for in the control samples, but both types were more frequent than the CHG type in all three samples. In the gene body region, similarly to previous studies in tomatoes and other eukaryotes, the CG type was found more frequently than the CHG and CHH types [9,21]. Furthermore, the methylation levels of the repeat regions were analyzed and the three methylation types showed similar distribution levels, with the methylation levels of the CG and CHG types being considerably higher than that of the CHH type.

As the DNA methylation status of the promoter and gene body regions could affect gene expression thorough changes in the chromatin structure or transcription efficiency [45,46,47], we compared the genome-wide methylation patterns of the control and sense-/antisense tomato fruit to identify the DMRs and investigated the biological functions of the DMRs and DEGs using the GO and KEGG analysis. Forty-three DEGs were found, of which 26 were upregulated and 17 were downregulated in the Sense_vs_Antisense group. As hypothesized, ERF1 was upregulated in the comparative analysis. As we know, the LeERF1 positively mediates ethylene signaling in tomato fruit [48]. In addition, several other ERFs showed different expression profiles. Intriguingly, most of the ethylene-related DEGs were of the CHH methylation type; however, CRF1, CRF2, and F-box proteins were involved in all three methylation types, and ARF16 was involved in the CG methylation type. This gives an idea of the specific roles of DNA methylation in the ethylene pathway (Figure 8, Table S6). 

The latest research results have shown that miRNAs direct DNA methylation at loci from which they are produced as well as in the trans region at their target genes. They also play important roles in gene regulation [49,50]. The first evidence for DNA methylation mediated by miRNA in the plant kingdom was reported in *Arabidopsis* [50,51]. In moss *Physcomitrella* patens, the ratio between miRNAs and their targets has been shown to activate miRNA-triggered DNA methylation [52,53]. It has also been previously reported that miRNAs mediate DNA methylation of their target genes [54]. In the current study, the relationships between the miRNA targets and DNA methylation levels and the different expressions of the miRNAs between different comparative groups were analyzed to determine where the target genes were involved in ARF16, ERF, ATHB, Lac, PPR, NB-ARC, and TIP. This indicated their specific roles in the ethylene pathway, opening a new window for the study of miRNA and DNA methylation co-regulation. 

## Supplementary Materials

The following are available online at http://www.mdpi.com/2073-4425/9/5/266/s1, Figure S1: Length distributions of the DMRs. Control vs Sense (J01 vs J02), Figure S2: Length distributions of the DMRs. Control vs Antiense (J01 vs J03), Figure S3: Length distributions of the DMRs. Sense vs Antiense (J02 vs J03), Table S1: The sample sequencing data evaluation statistics of the three samples, Table S2: The comparison results statistics of the three samples, Table S3: The detection of 5mC statistics of the three samples, Table S4: The GO and KEGG analysis results of DMRSs; Table S5: The DMRs and DEGs analysis results; Table S6: The DMRs and DEGs involved in ethylene pathway; Table S7: The relations between DNA methylation and miRNAs analysis results.
