# Supplementary material for: Comparative Analysis of DNA Methylation Reveals Specific Regulations on Ethylene Pathway in Tomato Fruit
Source: Genes (Basel). 2018 May 21;9(5):266. doi: 10.3390/genes9050266 (PMC5977206; doi:10.3390/genes9050266)

**Table S1.** The sample sequencing data evaluation statistics of the three samples

|  | **Clean_Reads** | **Clean_Base** | **GC(%)** | **Q20(%)** | **Q30(%)** |
| --- | --- | --- | --- | --- | --- |
| Control | 123648327 | 37196926100 | 22.44 | 95.78 | 90.23 |
| Sense | 132333959 | 39808257544 | 22.03 | 95.85 | 90.11 |
| Antisense | 142236149 | 42629605530 | 22.00 | 96.00 | 90.57 |

**Table S2.** The comparison results statistics of the three samples

|  | **Clean_Reads** | **Unique_mapped** | **Mapped(%)** | **Conversion_rate(%)** |
| --- | --- | --- | --- | --- |
| Control | 123648327 | 99968529 | 80.85 | 99.69 |
| Sense | 132333959 | 108317474 | 81.85 | 99.67 |
| Antisense | 142236149 | 116342830 | 81.80 | 99.67 |

**Table S3.** The detection of 5mC statistics of the three samples

|  | **mCHG** | **mCHH** | **mCpG** | **mC_total** | **mCHG(%)** | **mCHH(%)** | **mCpG(%)** |
| --- | --- | --- | --- | --- | --- | --- | --- |
| Control | 110297924 | 163323266 | 141715802 | 415336992 | 52.5 | 10.7 | 71.6 |
| Sense | 115773532 | 192161196 | 150413077 | 458347805 | 53.0 | 11.7 | 72.8 |
| Antisense | 116762764 | 206527819 | 151254752 | 474545335 | 52.6 | 12.5 | 72.3 |

**Figure S1.** Length distributions of the DMRs. Control vs Sense (J01 vs J02)


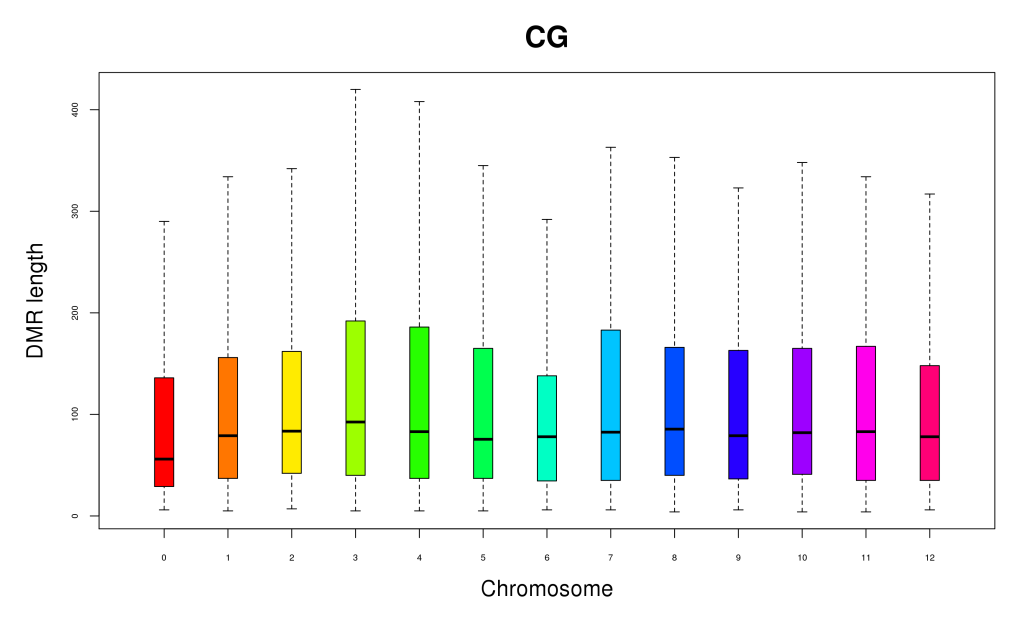


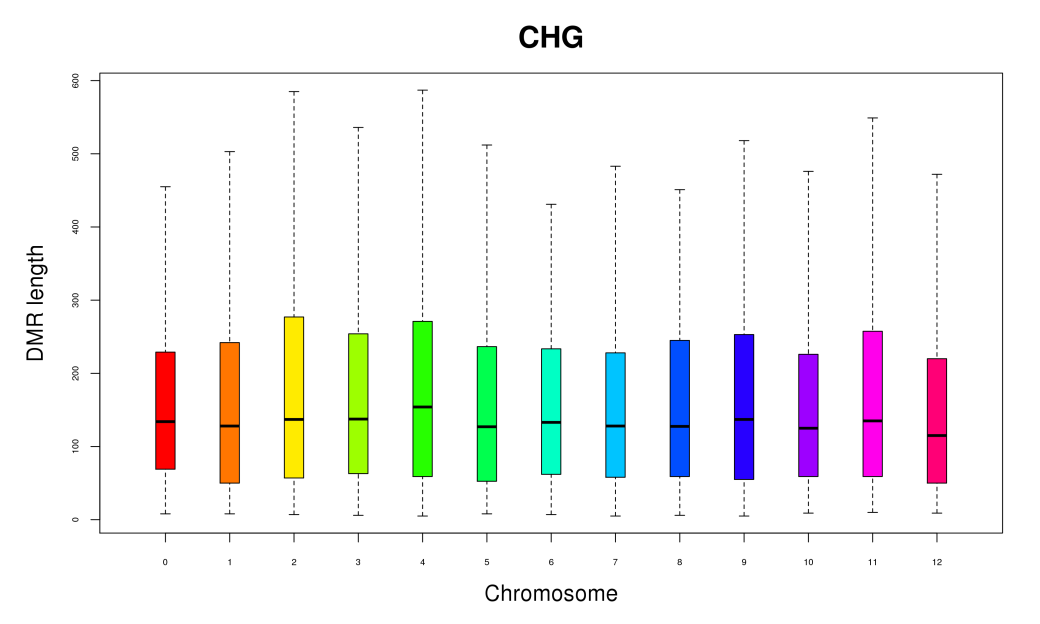


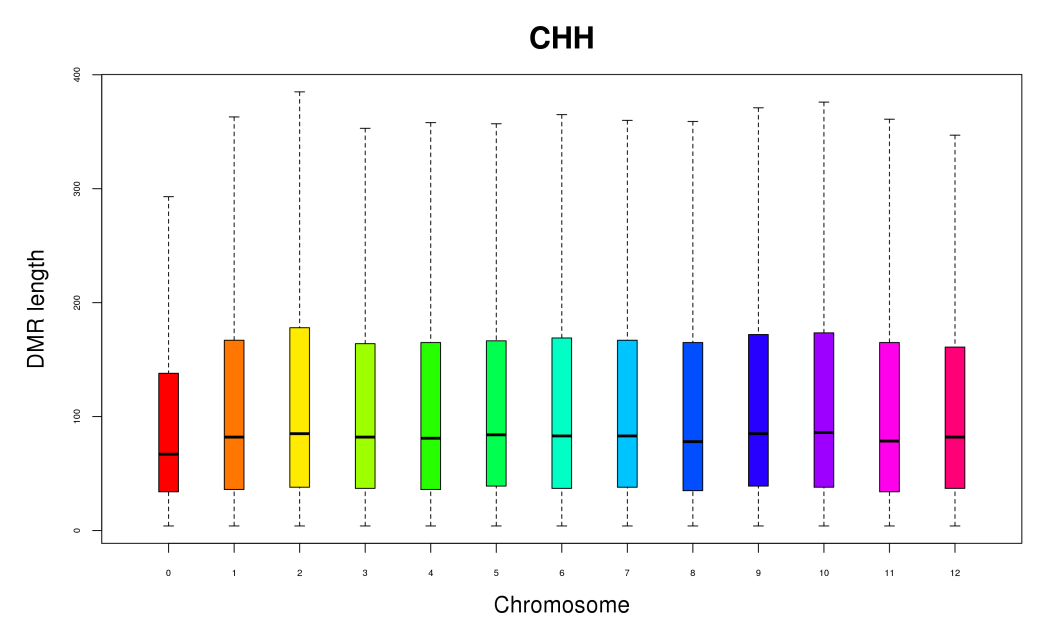


**Figure S2.** Length distributions of the DMRs. Control vs Antiense (J01 vs J03)


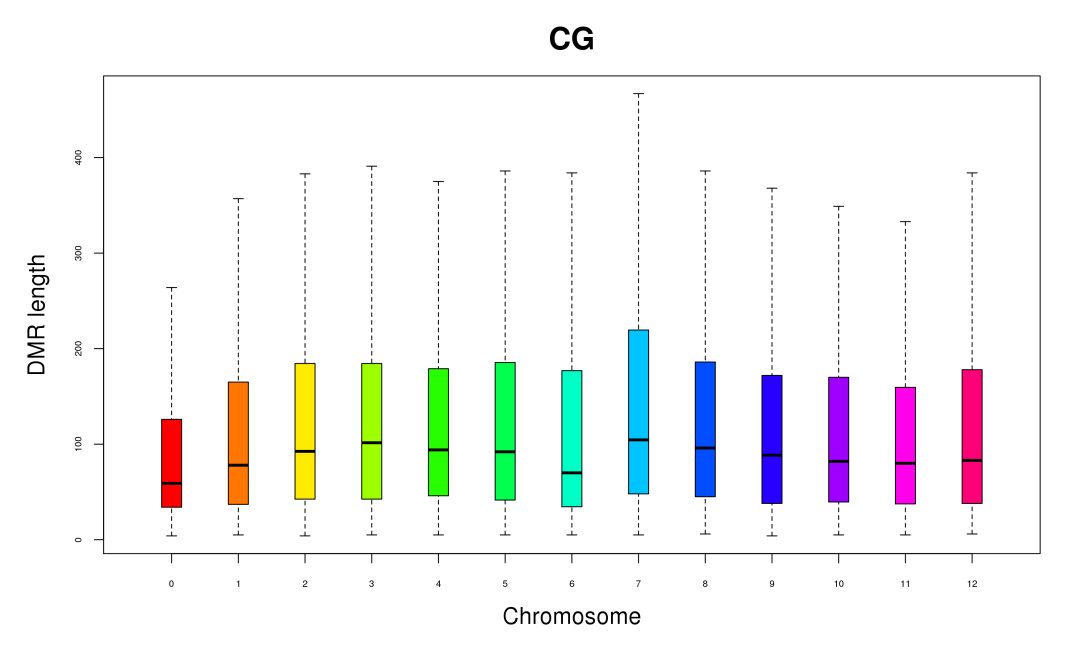


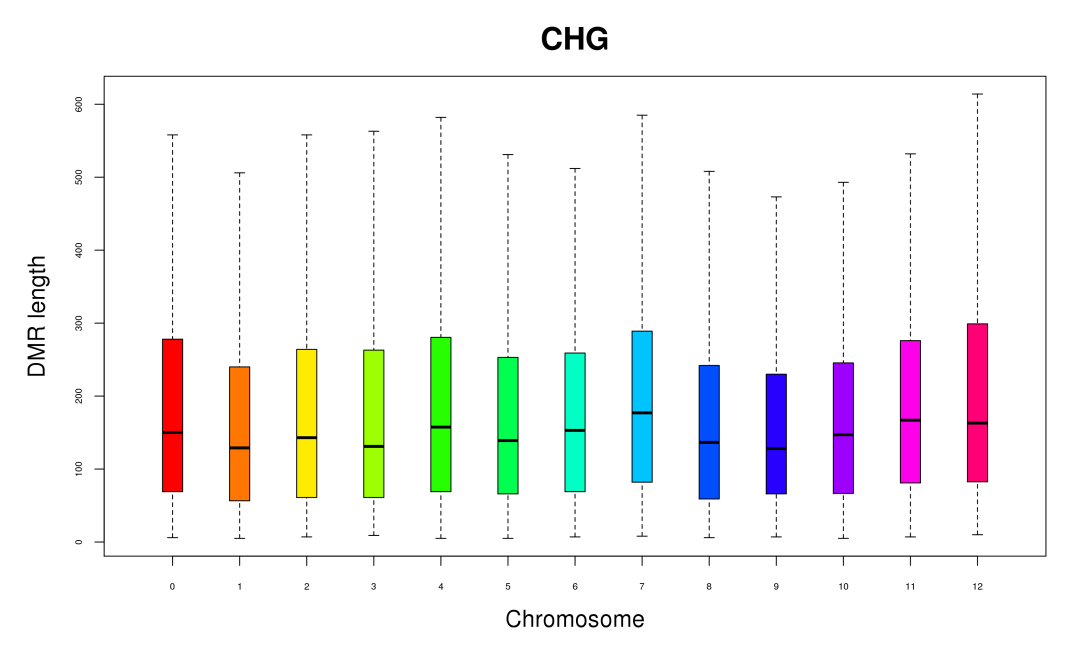


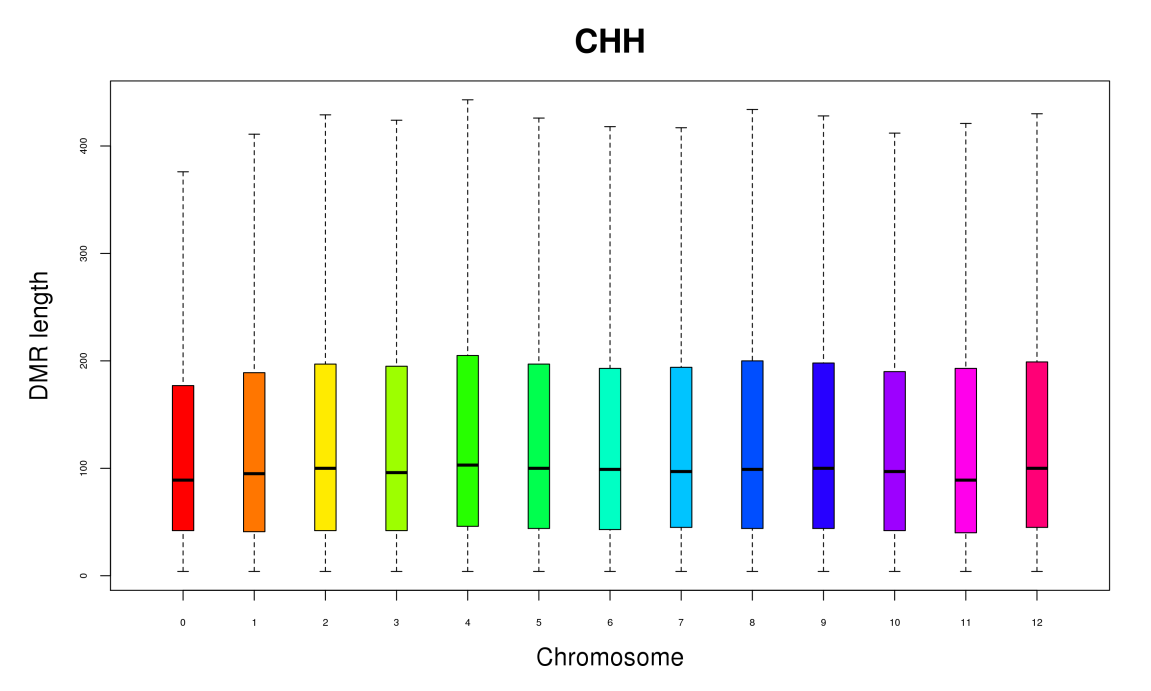


**Figure S3.** Length distributions of the DMRs. Sense vs Antiense (J02 vs J03)


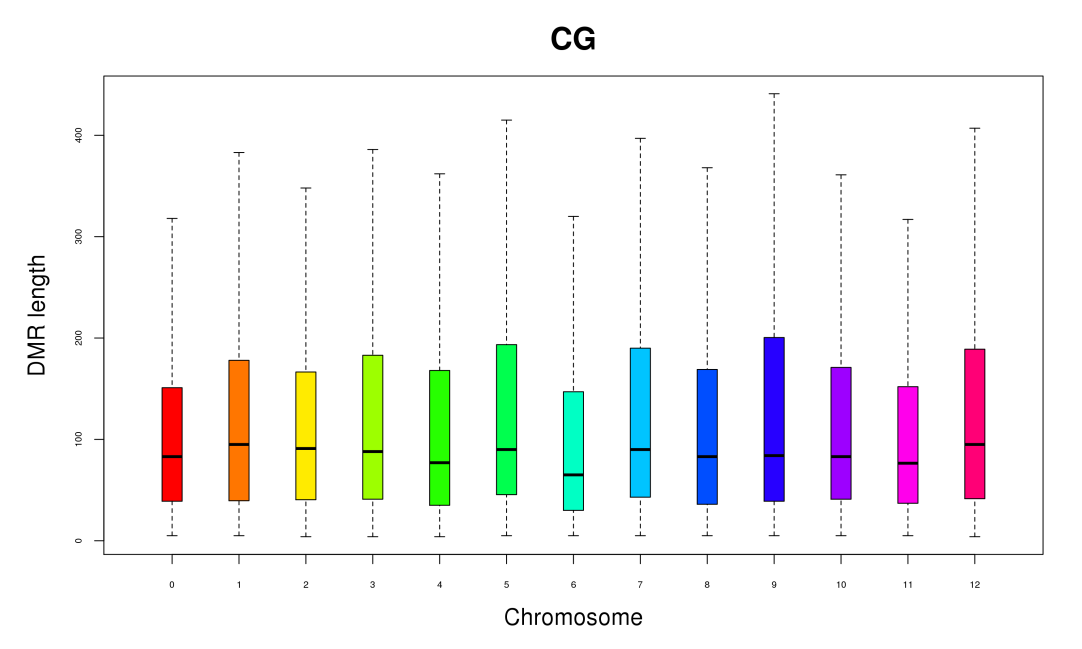


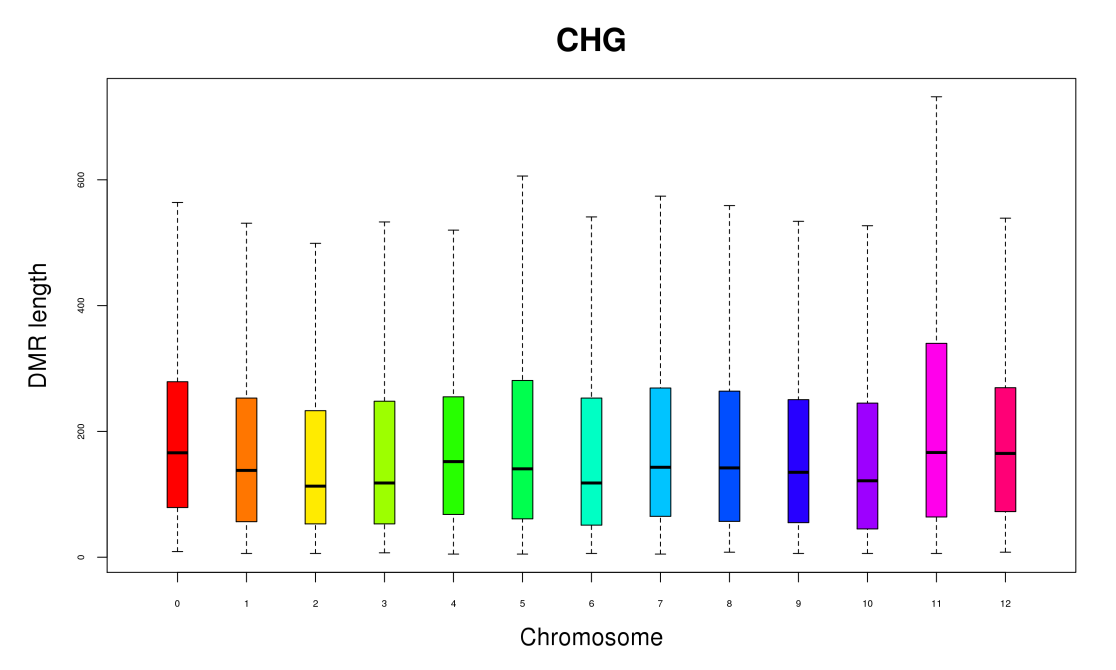


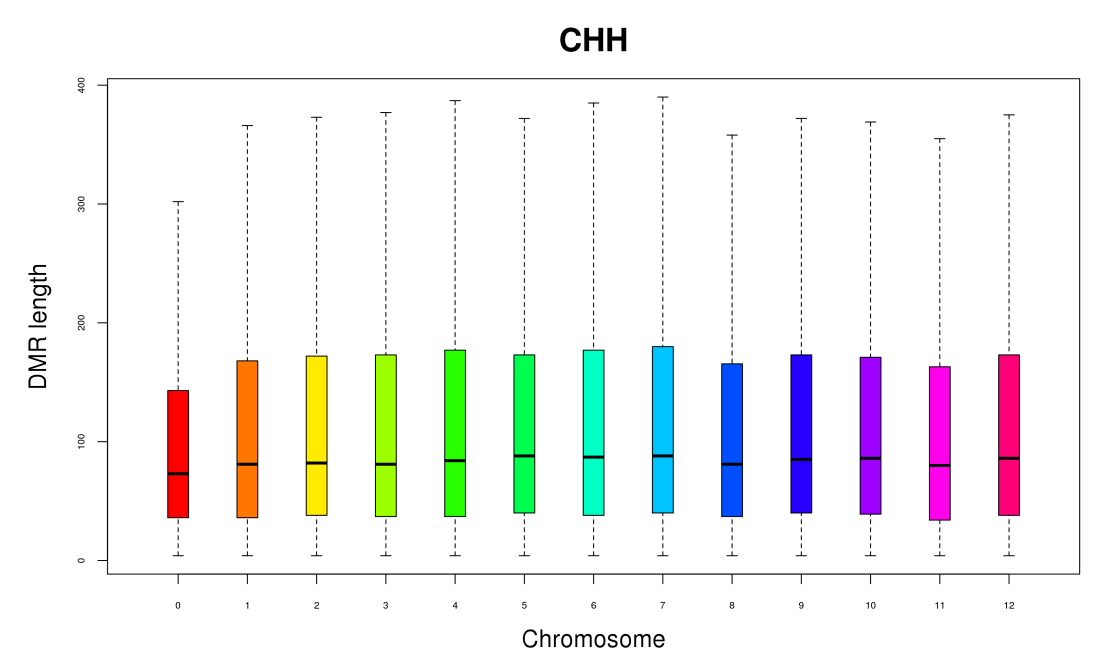

Supplement: Supplementary file 1 [file genes-09-00266-s001.zip › genes-294226-supplementary-final/Supplementary Tables S1-S3 - Figures S1-S3.docx]
